# Early Postpartum Maternal Morbidity among Rural Women of Rajasthan, India: A Community-based Study

**DOI:** 10.3329/jhpn.v30i2.11316

**Published:** 2012-06

**Authors:** Kirti Iyengar

**Affiliations:** Action Research and Training for Health (ARTH), Udaipur, India

**Keywords:** Anaemia, Community-based studies, Delivery, Morbidity, Mortality, Perinatal mortality, Postpartum care, India

## Abstract

The first postpartum week is a high-risk period for mothers and newborns. Very few community-based studies have been conducted on patterns of maternal morbidity in resource-poor countries in that first week. An intervention on postpartum care for women within the first week after delivery was initiated in a rural area of Rajasthan, India. The intervention included a rigorous system of receiving reports of all deliveries in a defined population and providing home-level postpartum care to all women, irrespective of the place of delivery. Trained nurse-midwives used a structured checklist for detecting and managing maternal and neonatal conditions during postpartum-care visits. A total of 4,975 women, representing 87.1% of all expected deliveries in a population of 58,000, were examined in their first postpartum week during January 2007–December 2010. Haemoglobin was tested for 77.1% of women (n=3,836) who had a postnatal visit. The most common morbidity was postpartum anaemia—7.4% of women suffered from severe anaemia and 46% from moderate anaemia. Other common morbidities were fever (4%), breast conditions (4.9%), and perineal conditions (4.5%). Life-threatening postpartum morbidities were detected in 7.6% of women—9.7% among those who had deliveries at home and 6.6% among those who had institutional deliveries. None had a fistula. Severe anaemia had a strong correlation with perinatal death [p<0.000, adjusted odds ratio (AOR)=1.99, 95% confidence interval (CI) 1.32-2.99], delivery at home [p<0.000, AOR=1.64 (95% CI 1.27-2.15)], socioeconomically-underprivileged scheduled caste or tribe [p<0.000, AOR=2.47 (95% CI 1.83-3.33)], and parity of three or more [p<0.000, AOR=1.52 (95% CI 1.18-1.97)]. The correlation with antenatal care was not significant. Perineal conditions were more frequent among women who had institutional deliveries while breast conditions were more common among those who had a perinatal death. This study adds valuable knowledge on postpartum morbidity affecting women in the first few days after delivery in a low-resource setting. Health programmes should invest to ensure that all women receive early postpartum visits after delivery at home and after discharge from institution to detect and manage maternal morbidity. Further, health programmes should also ensure that women are properly screened for complications before their discharge from hospitals after delivery.

## INTRODUCTION

Little rigorous research has been conducted on maternal morbidity in low-resource settings. Most studies are hospital-based, and if community-based, these tend to be retrospective and are based on self-reported symptoms, which are unreliable for estimating the prevalence of specific morbidities in the population. One study in the Philippines that validated self-reporting of obstetric complications found that the sensitivity of recall of haemorrhage, dystocia, sepsis, and eclampsia was 70%, 69%, 89%, and 44% respectively (1).

The early postpartum period is a time of heightened risk for both mothers and newborns. While significant progress has been made in developing community-based approaches for promoting neonatal health, similar attention has not been paid to improving maternal health during the postpartum period.

India witnesses the largest number of maternal and neonatal deaths in any single country, with over 63,000 maternal deaths and over one million neonatal deaths per year (2,3). Within India, Rajasthan has among the highest rates of maternal and neonatal death. Rajasthan is a large state in India, with a population of 68.6 million, 75% of which lives in rural areas (4). It has a high maternal mortality ratio of 318 per 100,000 livebirths (5) and a high neonatal mortality rate of 44 per 1,000 livebirths (6). Most maternal and neonatal deaths occur in the first seven days after delivery.

To develop standard integrated interventions for mothers and newborns, a sound understanding of conditions affecting both is essential. Action Research and Training for Health (ARTH), a non-governmental organization, aimed to develop an intervention to reduce maternal and neonatal mortality and morbidity in its rural field area with a population of 58,000 by providing integrated care to mothers and newborns within the first week after delivery. Services under this intervention are provided by trained nurse-midwives to all women and newborns, irrespective of the place of delivery. This paper presents the findings on the prevalence of various early postpartum maternal and neonatal conditions examined at home by trained nurse-midwives in a rural interior area in southern Rajasthan, India.

## MATERIALS AND METHODS

Since 1997, ARTH has implemented a field-level health service programme in a rural population of 58,000 in southern Rajasthan. Its field area comprises 49 villages surrounding two health centres that provide 24-hour delivery and newborn-care services through nurse-midwives. This intervention has been described earlier in details (7). Southern Rajasthan is hilly, and villages are scattered across several hamlets. While most villages are linked to roads, several hamlets are situated up to 3-4 km from the main village.

ARTH's village health workers (VHWs), the Government's accredited social health activists (ASHAs), and key-informants provide reports of all deliveries in the field area to the health centres, irrespective of the place of delivery. At each health centre, two or three nurse-midwives (trained auxiliary nurse-midwives–ANMs and general nurse-midwives–GNMs) provided reproductive and child-health services, including 24-hour delivery service and maternal and neonatal care while one nurse-midwife visits homes of all women as soon as possible after receiving the reports of deliveries.

### Collection of data

The programme started in April 2006. During April-December 2006, we developed the intervention, including pretesting and finalizing the structured examination checklist, training of nurse-midwives, developing formats for reporting of births, identifying village-level personnel to report births and orienting them, and developing data-management systems.

Forty-five ASHAs and six VHWs residing in the field area of ARTH were trained over two days to register all pregnant women, motivate them to seek at least four antenatal check-ups, and report all births in the field area, irrespective of the place of delivery. The ASHAs and VHWs visited each village and sought information about all recent births. In places where the ASHAs or VHWs were not active, various other persons, such as traditional birth attendants, jeep-drivers, and prominent women in the village, were identified as key-informants. They reported all deliveries in these 49 villages to the nearest ARTH health centres by telephoning at the centre or travelling there. They received an incentive for reporting deliveries early—Rs 50 (US$ 1) if reported within 24 hours, Rs 40 (US$ 0.8) if reported between 24 and 72 hours, and Rs 10 (US$ 0.2) if reported after seven days. Family members were also motivated through personal contacts, posters, and wall-paintings to inform about delivery. If the family reported a delivery, they were provided a free set of clothes for the newborn baby (costing ~Rs 40).

Six nurse-midwives employed in the ARTH's health centres were given a six-day additional training on postpartum maternal and neonatal care. The pre-service training of these nurse-midwives included either an 18-month course for ANMs or a three-year diploma in general nursing and midwifery. A structured checklist was developed and translated into Hindi for use by the nurse-midwives to detect complications in mothers and newborns as early as possible and manage these problems.

After the information on delivery was recorded, a nurse-midwife visited the home of the woman who recently delivered; the first visit was made as early as possible after delivery, preferably within 2-3 days after delivery and the second visit at 6-9 days after delivery. Arrangements were made to provide a transport to the nurse-midwives through a motorcycle for reaching the home of women since homesteads in the area are very scattered. The nurse-midwife used the detailed structured checklist to inquire about each condition of the mother and the baby and carried out a detailed physical examination. Examination of the mother included general examination, examination of breasts and abdomen, and a haemoglobin test at home using Sahli's method. Perineal and pelvic examinations were carried out only if the woman reported a symptom relating to these areas since these examinations were carried out in the home setting. Table 1 shows the activities carried out by the nurse-midwives to detect and manage postpartum maternal and neonatal complications, and the diagnostic criteria for various conditions are shown in Table 2.

If a woman or a newborn was detected to have a complication, the nurse-midwife treated it or, if severe, advised referral and informed about arrangements of free transport and treatment. Referrals were made either to the ARTH's RCH centres or to a referral hospital in the city, depending on the severity of the condition.

**Table 1.** Activities carried out by nurse-midwives during postpartum visits

Mother	Newborn
Detailed structured questionnaire, including that for postpartum depression and maternal morbidities	Enquiry about problems, using a structured checklist
Examination	Examination
General examination, including temperature, pulse, blood pressure, and respiratory rate Haemoglobin test for anaemia Breast and abdominal examinations Perineal and pelvic examination, if any complaint relating to these areas	Physical examination, including temperature, respiratory rate Weight Observation for local infections in eyes, umbilicus Examination for sepsis
Counselling and information on:	Counselling and information of mother on:
Diet and work	Breastfeeding
Danger signs and where to go for care	Bathing, keeping the baby warm Danger signs
Medications as per condition	Medications as per condition
Referral support	Referral support

**Table 2.** Diagnostic criteria for various morbidities

Condition	Diagnostic criteria	Test
Secondary PPH	Woman reports excessive bleeding 24 hours after childbirth and nurse-midwife finds the bleeding excessive on examination of pads	Structured questionnaire and examination of pad
Fever	Temperature above 38 °C	Measuring temperature
Uterine infection	Temperature above 38 °C and one of the following symptoms: Pain in lower abdomen Abnormal vaginal discharge Uterus not contracted well History of heavy vaginal bleeding	Clinical examination
Upper urinary tract infection	Temperature above 38 °C and one of the following symptoms: Burning in micturition Flank pain	Clinical examination
Other fevers	Temperature above 38 °C and none of the following symptoms: Pain in lower abdomen Abnormal vaginal discharge Uterus not contracted well History of heavy vaginal bleeding Burning in micturition Flank pain Breast pain	
Lower UTI	Burning in urination without fever	
Hypertensive disorders		Sphygmomanometry
Hypertension	Diastolic BP between 90 and 110 mmHg on 2 readings	
Severe hypertension	Diastolic BP >110 mmHg on 2 readings	
Anaemia		Haemoglobin testing at home, using Sahli's method
No anaemia	Hb 11.1 g or above conducted using Sahli's method	
Mild anaemia	Hb 9.1-11 g	
Moderate anaemia	Hb 7.1-9 g	
Severe anaemia	Hb 7 g or less	
Perineal conditions		Clinical examination
Perineal swelling	Excessive swelling of perineum or vulva on examination of perineum	
Perineal infection	Pain and pus or redness on examination of perineum	
Perineal pain	Woman reports pain in perineum	
Perineal tear	Tear in perineum on examination of perineum	
Breast-related conditions		Clinical examination
Breast engorgement	Engorged breasts, improper attachment of baby's mouth to breast and no fever	
Breast infection	Redness and swelling on one part of breast and one of the following: Woman feels sick Fever (temperature >38 °C)	
Flat or retracted nipple	Nipples are retracted or flat	
Postpartum blues	One of the following symptoms for less than 14 days: Cries easily Decreased interest or desire Feels tired or irritable all the time Unable to sleep Lack of appetite Diminished ability to concentrate	
Other infections		Structured questionnaire
Structured questionnaire
Respiratory infection with fever	Fever, cough, and chest-pain	
Upper respiratory infection	Cough and running nose without fever	
Tuberculosis	Woman is taking medicines for TB or cough for >3 weeks and blood in sputum and fever	
Incontinence and fistula		
Urinary incontinence	Woman reports frequent leaking of urine	Structured questionnaire
Fistula	Woman reports leaking of urine with confirmation on pad test and speculum examination	Clinical examination

BP=Blood pressure; Hb=Haemoglobin; PPH=Postpartum haemorrhage; TB=Tuberculosis; UTI=Urinary tract infection

To assess the quality of care at postnatal visits, a senior nurse-midwife or a physician accompanied the nurse-midwife during 5% of postnatal visits and assessed the quality of care provided. A research manager also visited 70% of women at 4-8 weeks after delivery and made enquiries, using a standard checklist regarding some procedures, such as measuring blood pressure, haemoglobin, weighing of the newborn, and counselling. If any gap in care was detected, they immediately gave feedback to the nurse-midwives. Additionally, the intervention was discussed once a month among the researchers, supervisors, and some of the nurse-midwives. The nurse-midwives could not be called for all meetings since they were in the health centres or in the field, and their attendence in a meeting would mean disruption of 24 × 7 delivery service or postnatal visits for that day. The feedback of the nurse-midwives was sought frequently during field-visits by the project personnel.

**Fig. 1.**
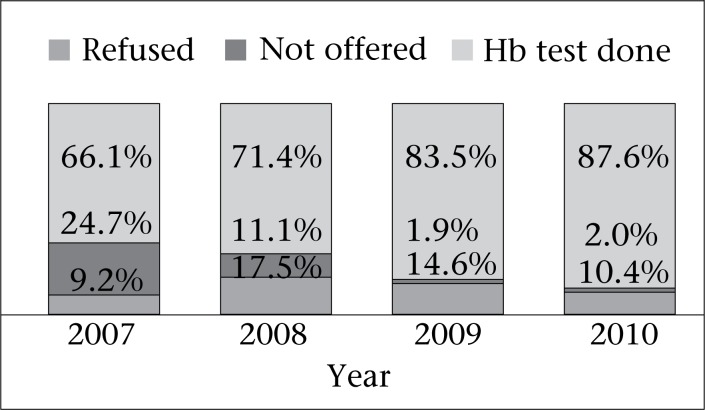
Haemoglobin testing during postnatal home-visit

On visiting a woman's home, the nurse-midwife informed her of the purpose of the visit and sought verbal consent for interview and examination. A minority (1.1%) of women visited by the nurse-midwives refused the postnatal check-up. During examination, 12.8% of the women refused haemoglobin test. For another 10.2% of the women, the nurse-midwives did not offer haemoglobin test. This happened particularly in the first year when nurse-midwives did not conduct a haemoglobin test if the woman had undergone a haemoglobin test during the antenatal period. Subsequently, during review meetings, the need to conduct the haemoglobin test during postpartum visits was emphasized, even if the woman had a recent haemoglobin test in the antenatal period. Hence, haemoglobin was eventually tested for 77.1% of women during postnatal visits (Fig. 1). Data were analyzed using the Epi Info and Stata software (version 11).

The implementation of the programme started in January 2007 and is continuing till date. In this paper, we are presenting data for the January 2007–December 2010 period.

## RESULTS

### Number of reported births and postnatal visits

Over a four-year period from January 2007 to December 2010, we expected 5,712 births in the field area based on the birth rate of 26 per 1,000 people. Of these, 5,266 deliveries (92.2% of the expected births in the community) were reported to the nurse-midwives within 28 days after delivery, and 5,042 births (88.3% of the expected deliveries) were reported within 14 days. The median interval between delivery and reporting was one day. Sixteen postpartum maternal deaths also occurred during the four-year period in this field area, nine of which occurred within seven days after birth. However, we did not present data on these maternal deaths in this paper as it focuses on morbidities detected during the postpartum visits.

**Fig. 2.**
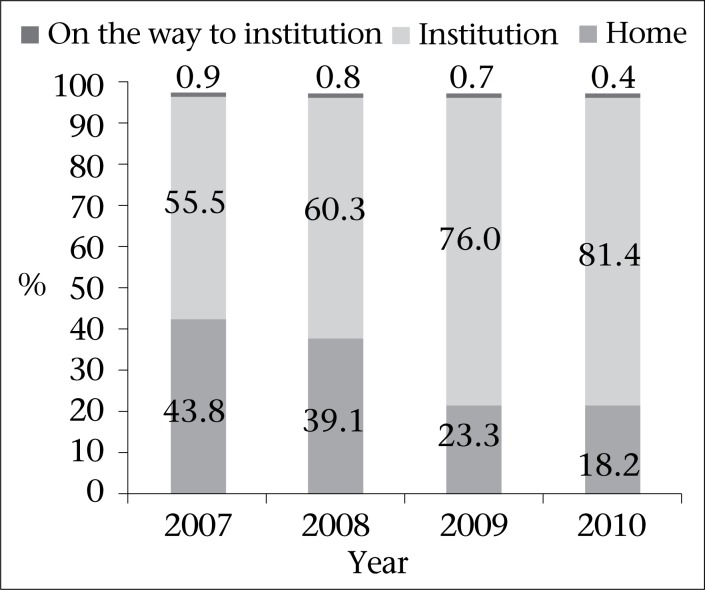
Place of reported deliveries over years

After the initiation of a national scheme called Janani Suraksha Yojana to provide cash incentives to women delivering in government institutions in 2006, there has been a major shift in the place of delivery from home to institutions—starting with 53% in 2007 and increasing to 82% in 2010. Figure 2 shows average data of the respective years. Overall, 68.2% of the reported deliveries occurred in institutions, 31.1% at home, and 0.7% on the way to the institutions.

The nurse-midwives made home-level postnatal visits to 4,975 women (94.5% of the women whose births were reported and 87.1% of the expected number of births in the area). In 5.5% of the women, the postnatal care (PNC) visits could not occur despite receiving a report of delivery. This was primarily due to the leave schedule of nurse-midwives: when more than one nurse-midwife was on leave at a health centre, 24 × 7 delivery service of the health centres received priority over postnatal visits. An attempt was made to ensure that the visit occurred as early as possible. The median interval between delivery and the first PNC visit was five days in the first year and three days in the fourth year, with an average of four days. In some cases, the PNC visits occurred late either because the delivery report came late, or the nurse-midwife or the motorcycle-driver was on leave, or because the two-wheeler broke down on a given day. In some cases, when the nurse visited the home on day 2 or 3 after an institutional delivery, the woman had not yet returned home. In such cases, the visit was made again after 2-3 days.

The large majority (78%) of the women were in their twenties, and 60.2% belonged to the socioeconomically-underprivileged scheduled tribes or scheduled castes (Table 3). For one-fourth of the women, this was their first delivery, and for nearly 30%, it was their fourth or subsequent delivery.

More than one-third of the women delivered at home while nearly 68.6% delivered in institutions ranging from village-level government subcentres to the district hospital, with 33.0% delivering in the ARTH's health centres. Only 2.2% delivered through caesarean section, and the remaining women had vaginal delivery. During January to December 2009, we recorded information on blood transfusion and episiotomy for all 1,156 births. It showed that 1.4% of the women received a blood transfusion in pregnancy or around the time of delivery, and 7.1% reported having an episiotomy.

### Types of maternal morbidities detected

Nearly three-fourths of the women were detected to have a morbidity after delivery. The most common problems were postpartum anaemia, sepsis, and breast and perineal infections (Table 4).

The most common serious morbidity detected was severe anaemia present in 7.4% of women whose haemoglobin was tested (5.7% of all women). Fever was present in 4.0% of the women, although signs of uterine infection were present in only 1.3% of the women. The remaining women with fever had an upper urinary tract infection or respiratory infections. The incidence of puerperal sepsis was 1.4% following home-delivery and 1.2% following institutional delivery. The incidence of any kind of infective illness after delivery was 6.0% following home-delivery and 5.7% following institutional delivery.

Conditions relating to breasts (breast engorgement, mastitis, or flat nipple) were detected in 4.9% of the women—none, however, had a breast abscess on the day of the postnatal visit. Breast infections were also more frequent among women who had institutional deliveries. Additionally, breast conditions were more common among women with perinatal death than among those with a surviving neonate (13.1% and 4.3% respectively). Conditions relating to the perineum (perineal pain, tear, or infection) were detected in 4.5% of the women. The prevalence of perineal conditions was significantly more frequent among women who had institutional deliveries (6%) than among those who had home-delivery (1.1%). On further analysis for the one-year period for which there were data on episiotomy, we found that the incidence of any perineal condition was 28% among those with an episiotomy compared to 3.0% of those without episiotomy.

**Table 3.** Profile of women and characteristics of delivery of women to whom postnatal care was provided

Background characteristics	No. of women	%
Age (years)	(n=4,975)	
15-19	88	1.8
20-29	3,889	78.2
30-39	977	19.6
40-49	21	0.4
Caste	(n=4,975)	
Scheduled tribe or caste*	2,994	60.2
Other	1,981	39.8
No. of children		
1	1,303	26.2
2-3	2,167	43.6
4-5	1,144	23.0
6 and above	361	7.3
Place of delivery	(n=4,975)	
Home	1,530	30.8
On the way to the institution	33	0.7
Institution	3,412	68.6
Government's subcentre/PHC/CHC	941	18.9
District hospital	827	16.6
NGO health centre	1,644	33.0
Mode of delivery	(n=4,975)	
Caesarean section	107	2.2
Vaginal delivery	4,868	97.8
Women with blood transfusion and episiotomy	(n=1,156)	
% received blood transfusion	16	1.4
% who had episiotomy	82	7.1

*Scheduled castes and tribes are socially- and economically-marginalized groups as listed in the Constitution of India; CHC=Community Health Centre; PHC=Primary Health Centre

**Table 4.** Postpartum maternal morbidities detected by nurse-midwives (n=4,975)

Type of maternal problems	Total (%)	Home-delivery (n=1,560)	Institutional delivery (n=3,415)
Secondary PPH	21 (0.4)	7 (0.4)	14 (0.4)
Fever	200 (4.0)	58 (3.7)	142 (4.2)
Uterine infection	64 (1.3)	22 (1.4)	42 (1.2)
Upper urinary tract infection	28 (0.6)	6 (0.4)	22 (0.6)
Pneumonia	35 (0.7)	9 (0.6)	26 (0.8)
Only fever	73 (1.5)	21 (1.3)	52 (1.5)
Other infections			
Upper respiratory infection	94 (1.9)	34 (2.2)	60 (1.8)
Tuberculosis	6 (0.1)	1 (0.1)	5 (0.1)
Lower urinary tract infection	151 (3.0)	38 (2.4)	113 (3.3)
Hypertensive disorders			
Moderate hypertension	50 (1.0)	11 (0.7)	39 (1.1)
Severe hypertension	7 (0.1)	0 (0.0)	7 (0.2)
Anaemia (3,836 women in whom Hb test was done)			
Severe	285 (7.4)	123 (11.6)	162 (5.8)
Moderate	1,755 (45.8)	483 (45.4)	1,272 (45.9)
Mild	1,590 (41.4)	393 (36.9)	1,197 (43.2)
Perineal conditions	223 (4.5)	15 (1.0)	208 (6.1)
Perineal swelling	34 (0.7)	3 (0.2)	31 (0.9)
Perineal pain	140 (2.8)	9 (0.6)	131 (3.8)
Perineal infection	21 (0.4)	2 (0.1)	19 (0.6)
Perineal tear	29 (0.6)	2 (0.1)	27 (0.8)
Breast-related conditions	242 (4.9)	50 (3.2)	192 (5.6)
Breast engorgement	146 (2.9)	34 (2.2)	112 (3.3)
Breast infection	63 (1.3)	10 (0.6)	53 (1.6)
Retracted nipple	33 (0.7)	6 (0.4)	27 (0.8)
Postpartum blues	70 (1.4)	28 (1.8)	42 (1.2)
Long-term conditions			
Urinary incontinence	4 (0.1)	1 (0.1)	3 (0.1)
Fistula	0 (0)	0	0
Other (including diarrhoea, vomiting, and other conditions)	39 (0.8)	7 (0.4)	32 (0.9)
At least one of the morbidities (including all the above conditions, except mild anaemia)	3,364 (67.6)	1,107 (71.0)	2,257 (66.1)
Life-threatening conditions (severe anaemia, puerperal sepsis, severe hypertension, and secondary PPH)	376 (7.6)	152 (9.7)	224 (6.6)
Any infective illness (uterine infection, upper UTI, lower UTI, pneumonia, only fever, perineal infection, breast infection, TB, upper respiratory infection)	288 (5.8)	93 (6.0)	195 (5.7)

Hb=Haemoglobin; PPH=Postpartum haemorrhage; TB=Tuberculosis; UTI=Urinary tract infection

**Fig. 3.**
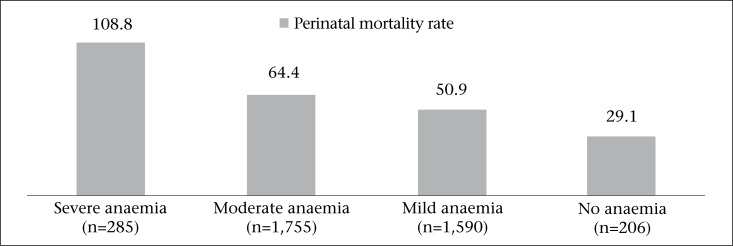
Correlation between severity of anaemia and perinatal mortality

Urinary incontinence was reported by 0.1% of the women. None of the women had genito-urinary fistula. Life-threatening complications, such as severe anaemia, uterine infection, secondary postpartum haemorrhage (PPH), and severe hypertension or eclampsia, were experienced by at least 7.6% of the women, of which 5.7% had severe anaemia, and 1.8% had one of the other conditions. Since haemoglobin was not tested in 22% of the women, it is possible that the actual prevalence of life-threatening conditions was higher. Life-threatening conditions were present in 9.7% of those who had home-deliveries and 6.6% in those who had institutional deliveries. A large proportion (28%) of the women also reported lower abdominal pain, backache, or pain in arms and legs.

**Table 5.** Correlations of severe anaemia

Condition	% with severe anaemia	p value	Crude odds ratio	Adjusted odds ratio (95% CI)*
Caste				
Scheduled caste or tribe (n=2,326)	9.7	<0.000	2.65 (1.98-3.56)	2.47 (1.83-3.33)
Other (n=1,510)	3.9			
Place of delivery				
Home (n=1,067)	11.6	<0.000	2.13 (1.67-2.72)	1.64 (1.27-2.15)
Institution (n=2,769)	5.8			
Number of children ever born				
1-2 (n=2,198)	4.9			
3 or more (n=1,638)	10.9	<0.000	1.83 (1.42-2.35)	1.52 (1.18 −1.97)
Whether received ANC				
Received ANC (n=3,565)	7.3	0.362	0.82 (0.52-1.26)	1.12 (0.71-1.76)
No ANC (n=271)	8.9			
Child outcome				
Perinatal death (stillbirth + early neonatal death) (n=232)	13.4	<0.000	2.03 (1.36-3.03)	1.99 (1.32-2.99)
No perinatal death (n=3,604)	7.0			

*Adjusted for caste, parity, and place of delivery; ANC=Antenatal care; CI=Confidence interval

Since anaemia was the most common postpartum morbidity among the women, we looked at correlations for severe anaemia (Table 5). We found that severe anaemia was more prevalent among women who had home-deliveries and among women from the scheduled castes and tribes. Furthermore, multiparous women (having 3 or more children) were more likely to have severe anaemia than those with 1-2 child(ren). It was, however, not significantly different between women who received antenatal care and those who did not. We do not have information on whether women consumed iron during pregnancy or not or whether they had anaemia before delivery.

The severity of anaemia had a linear correlation with perinatal mortality (Fig. 3). Compared to women with no anaemia, women with severe anaemia were 3.7 times more likely to have a perinatal death while compared to mild anaemia, they were 2.1 times more likely to have perinatal death. The differences were significant [p<0.000, OR=2.35 (95% 1.53-3.61)]. These differences remained significant even after controlling for caste, parity, and place of delivery [AOR=2.36 (95% CI 1.5-3.69)].

## DISCUSSION

This study adds valuable knowledge on postpartum morbidity affecting women in the first few days after delivery in a low-resource rural setting. The results of the study showed that, during the first week after delivery at home or institutional delivery, women suffered a high burden of morbidities.

The most common postpartum maternal morbidities were moderate and severe anaemia in our study. While many studies have assessed the prevalence of anaemia in the antenatal period, very few have assessed it in the postpartum period. In one study in rural Bangladesh, more than 10% of women were severely anaemic in the postpartum period, at both 48 hours after delivery and two weeks later (8). A cross-sectional survey in Viet Nam found that the prevalence of anaemia in the postpartum period was higher among pregnant women (9). A community-based study from north India has shown that 70% of women were anaemic at six weeks postpartum (10). Studies from high-income countries have shown a lower prevalence of postpartum anaemia (11). The high prevalence of anaemia in our study is not surprising as it has long been reported that South-East Asia has the highest prevalence of anaemia among pregnant women in the six regions of the World Health Organization (WHO) (12).

Given these findings, it is important to address anaemia in the postpartum period and prenatally because it contributes to maternal deaths both directly and indirectly—acute onset of anaemia can lead to rapid cardiac decompensation and heart failure (13). It also aggravates the effects of sepsis and haemorrhage—in the latter, anaemia puts women at risk of hypotension and death with even moderate bleeding. Studies in India have found a high proportion of maternal deaths attributable to anaemia which was reported to be a cause in almost one-fifth (19.0%) of maternal deaths in rural India (14) while the WHO's analysis of causes of maternal deaths stated that anaemia contributed to 12.8% of all maternal deaths in Asia (15). One hospital study in Rajasthan reported that anaemia contributed to 24% of all maternal deaths (16). Results of a verbal autopsy study in rural Rajasthan showed that anaemia was the second biggest cause of postpartum maternal deaths, responsible for 26.3% of postpartum maternal deaths (17), and all deaths due to anaemia occurred in the postpartum period.

Although controlling antenatal anaemia is likely to reduce the prevalence of postpartum anaemia, there is still a need to detect and manage postpartum anaemia because anaemia can result or worsen as a result of blood loss during delivery. According to the Global Burden of Disease 2003, postpartum anaemia is the most important consequence of postpartum haemorrhage (18). The report estimated that the incidence of PPH (defined as >1,000 mL of blood loss within one hour postpartum) was 2.9% in women whose third stage was actively managed using oxytocin, 5.7% among those who were managed expectantly by a skilled birth attendant, and 11.4% among births without skilled attendance (18).

Women who are moderately anaemic in pregnancy are likely to become severely anaemic if they had blood loss of even moderate amounts during delivery. Since very few of these women in low-resource settings are likely to receive treatment or blood transfusion after delivery, they are likely to remain severely anaemic and suffer from mortality or morbidity due to this. For example, only 1.3% of the women in our study received blood transfusion in their recent pregnancy, labour, or first week after delivery.

A recent meta-analysis showed that correcting anaemia of any severity was associated with reduced risk of death; for each gram percentage of increase in haemoglobin, the risk of death is reduced by 20% (19). However, very little attention is currently given to postpartum anaemia, and there is no programme to detect and manage postpartum anaemia in developing-country settings.

Our study showed a linear correlation between the level of postpartum anaemia and perinatal mortality. Results of a meta-analysis of health risks revealed that iron-deficiency anaemia was associated with 24% of perinatal deaths (19). Anaemia in the antenatal period leads to low birthweight and preterm births, which, in turn, contribute to perinatal death (20,21). However, it is not clearly established whether supplementation of iron in pregnancy reduces perinatal mortality (22).

In our study, severe anaemia positively correlated with home-delivery, multiparity, and scheduled tribe or caste. However, no significant difference was observed in rates of postpartum anaemia between women who received antenatal care and those who did not. This is not surprising since all women do not get iron supplementation, and even when they get, the compliance is poor. Only 53.6% of all pregnant women received iron supplementation for 100 days or more in Rajasthan (23).

In our study, fever was detected in 4.0% and puerperal sepsis in 1.3% of women. The incidence of puerperal sepsis in our study is comparable with findings of other studies, although most studies of puerperal sepsis are hospital-based. In a hospital-based study covering 75,497 women, endometritis was detected in 0.17% of women (24). Since most cases of sepsis develop after discharge, Yokoe and others followed a comprehensive post-discharge surveillance procedure method and noted that the incidence of sepsis following vaginal birth in facilities was 2.5% (25). In Nigeria and Malawi, three hospital-based studies showed an incidence of puerperal sepsis of 1.34-1.49% (26-28). Some studies have compared the rates of genital sepsis following births in the home and facility and reported higher rates of sepsis following home-deliveries (29,30).

In a community-based study in a rural midwestern Indian state, fever was detected in 8.9% of women by village health wokers on self-reports (31). Dolea and Stein estimated that the burden of maternal sepsis in six countries of the South-East Asian Region is 4.5 per 100 livebirths (32). In our study, the incidence of puerperal fever among women who delivered at home was 3.7% compared to 4.2% in those who delivered in facility. We feel that the higher incidence of fever after institutional deliveries could be related to sub-optimal aseptic conditions, such as multiple pelvic examinations (33). Since postpartum fever usually develops a few days after delivery, postpartum home-visits are necessary to detect and manage this condition.

During the first few weeks postpartum, although pain in the perineum and vulva is an important problem, research on this issue is scanty. In Egypt, 2.1% of women reported dyspareunia after childbirth (34). Some hospital-based studies have shown a much higher incidence of perineal pain after childbirth. One study in Canada found that 38% of women with intact perineum and 71% of women with episiotomies suffered from perineal pain seven days after childbirth (35). In addition, results of a study in Nigeria showed that 28% and 69% of women with intact perineum and those with episiotomy respectively had perineal pain three days after delivery (36). Our study found that 4.5% of the women had conditions relating to perineum (pain, tear, or infection) during the first week postpartum, and it was five times higher among women who had institutional deliveries. A relatively low prevalence of perineal pain observed in our study compared to the aforementioned studies could be due to the low incidence of episiotomy (7%). Episiotomy or perineal tears during childbirth are associated with significant pain, infection, and loss of mobility during the immediate postpartum period (37). The avoidance of unnecessary episiotomies can also reduce perineal pain (38) and infections. Currently, perineal pain is inadequately managed and needs greater attention.

Breast conditions (engorgements, infections, abscess, or retracted nipples) were detected in 4.9% women in our study, with 1.3% having mastitis. The prevalence of mastitis varies depending on the definition and the number of weeks postpartum (39); the highest incidence has been reported at four and 12 weeks. Mastitis is reported to occur in 2-24% of breastfeeding women from several weeks up to one year after delivery in women who continue to breastfeed (40). Results of studies from developed countries showed that the reported cumulative incidence of mastitis varies from a few to 33% of lactating women but it is usually below 10% (41). Estimates of incidence of breast abscess from developed countries showed that the incidence varies from 0.04% to 0.4% (42,43). Very few studies have been conducted on the incidence of mastitis in developing countries. The lower incidence of mastitis in our study could be due to two reasons: first, we collected data in the first week after delivery, and second, breastfeeding is nearly universal in our study area. It is crucial to manage breast conditions because women suffer from pain due to these conditions. Breast infections form a considerable burden of disease, involve substantial costs (44), and can occasionally be fatal if untreated (41). Furthermore, breast conditions are often a reason for stopping breastfeeding (41) and hence, a higher risk to the neonate.

We did not find any case of fistula in the 4,975 rural women. The incidence of fistula is not clearly known. It appears that there are important geographic variations in the prevalence of obstetric fistulae; specifically, it appears to be more common in sub-Saharan Africa than in other parts of the developing world (45). Many studies on fistulae are based on hospital-records, or on reports by gynaecologists or surgeons who provided information on the proportion of fistula cases from among total admissions. While they provide good indication of the existence of fistulae in particular areas of the world, they do not furnish adequate data on its true incidence. Reports from Nigeria have shown that about 1 in 1,000 deliveries is complicated by obstetric fistula (46). No comprehensive data on the epidemiological trends are available for the South Asian region. A survey conducted in 2003 to investigate the fistula situation in Bangladesh found that the number of fistula cases per 1,000 ever-married women was 1.69 (47). In a community-based study in rural India, no cases of fistula were reported when women were contacted in their homes during the first few days after delivery (31). The absence of fistula in our study is perhaps related to reduction in cases of prolonged obstructed labour because of improvement in the road network and increase in skilled attendance at delivery.

### Limitations

One limitation of our data was that haemoglobin was tested for only 77.1% of the women. Comparison of women whose haemoglobin was tested and not tested revealed that the proportion of women with home-deliveries was higher among those whose haemoglobin was not tested. Since the prevalence of postpartum anaemia was higher among women with home-deliveries in this study, the actual proportion of women with postpartum anaemia could be higher. Another limitation of the study was that we did not have information about women's problems prior to delivery.

### Conclusions

Life-threatening conditions, such as severe anaemia, puerperal sepsis, severe hypertension, and secondary PPH, were experienced by 7.6% of the study women. Since most women do not receive any postpartum care at the home level, there is a risk of maternal and late maternal death and long-term consequences for such women in the next few months. A large proportion of women also suffer from other less-serious morbidities, which take a toll on women's day-to-day performance. There is evidence that women with severe and less-severe maternal complications in the early postpartum period suffer from many physical, mental, social and economic consequences (48) and a higher risk of death and infant mortality. Hence, it is essential that health programmes make investments to provide postpartum care to all women starting from the first week so that these conditions can be detected and managed in time. This is especially important for those delivering at homes. However, even women who delivered in institutions suffered from many health problems, including life-threatening complications. This suggests that there is a need to screen all women properly before discharge from the facility. The results of our intervention also suggest that it is feasible for skilled birth attendants to visit women's homes and provide postpartum maternal and neonatal care to them in an integrated manner.

## ACKNOWLEDGEMENTS

This intervention was financially supported by John D. and Catherine T. MacArthur Foundation. The funder has had no involvement in the design of the intervention or conclusions drawn. The author thanks all the women who agreed to participate in this intervention and acknowledges the invaluable contribution of the nurse-midwives who carried out the postnatal home-visits.
